# Supplementary material for: Sulfation of O-glycans on Mucin-type Proteins From Serous Ovarian Epithelial Tumors
Source: Mol Cell Proteomics. 2021 Sep 21;20:100150. doi: 10.1016/j.mcpro.2021.100150 (PMC8527052; doi:10.1016/j.mcpro.2021.100150)
Supplement: Supplemental Figs. S1–S3 [file mmc1.pdf]

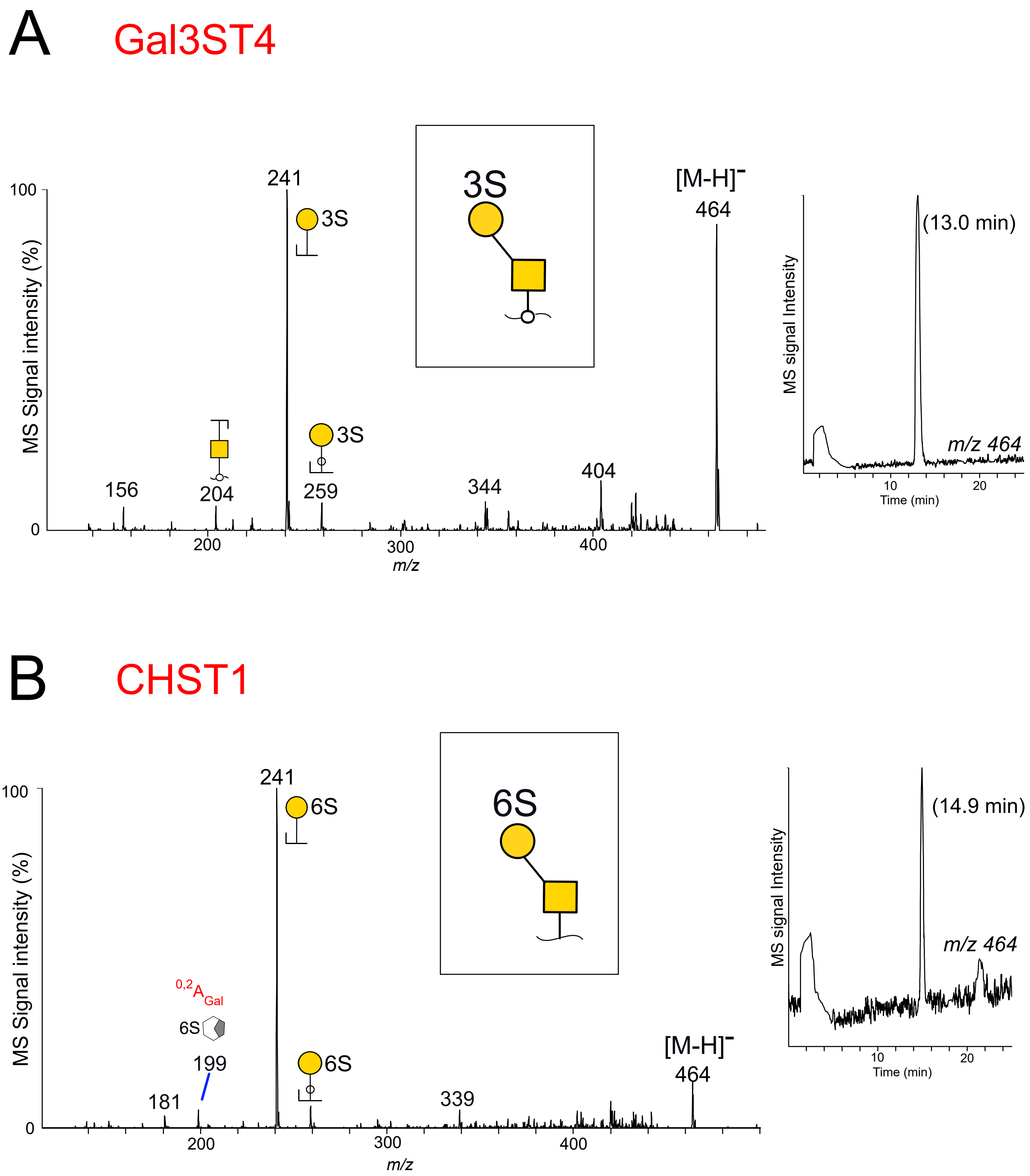

Supplemental Figure 1 LC- MS^2 spectra of sulfated core 1 O-glycans collected after fragmentation of the precursor ion at *m/z* 464 ([M-H]-) from recombinantly produced PSGL-1/mIgG2b protein purified from CHO cells. PSGL-1 was co-transfected with Gal3ST4 (A) or CHST1 (B). Included in each figure is EIC of *m/z* 464 from LC-MS run. For key of symbols, see Figure 1.

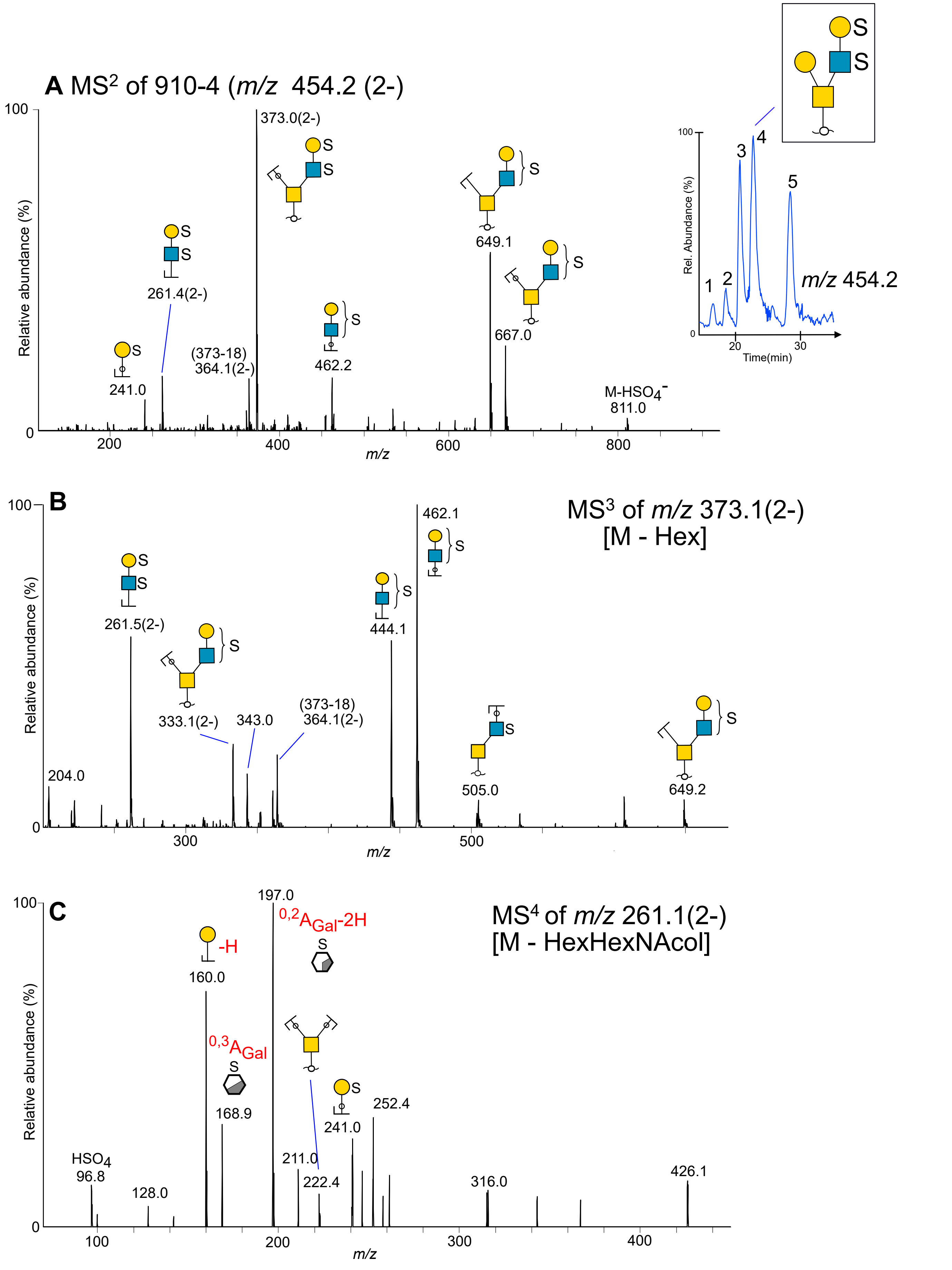

Supplemental Figure 2 Fragmentation of the disulfated isomer 910-4. Glycan sequence was interpreted from MS2, MS3 and MS4 experiments. (A) MS^2 spectrum of parent detected as $[M-2H]^{2-}$ ions at *m/z* 454.2^{2-} (inserted EIC). Fragment ions are annotated with proposed compositions. For some fragment ions, more than one composition may be possible. (B) MS^3 spectrum of the *m/z* 373.0^{2-} from the MS^2 experiment shown in (A). (C) MS4 spectra of the ions at *m/z* 261.1^{2-} from the MS^3 experiment shown in (B). For key of symbols, see Figure 1.

m/z *[M-xH]ˣ⁻*	PSGL-1/mIgG2b + GCNT1[1]	+ GAL3ST2	+ GAL3ST4	+ CHST1
675	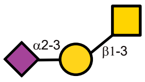 2%	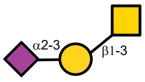 8%	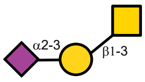 11%	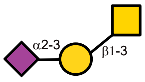 10%
749	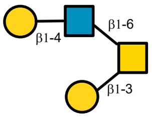 1%		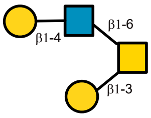 6%	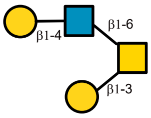 6%
878	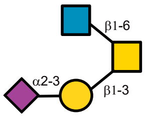 2%	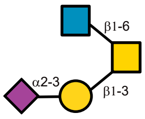 6%	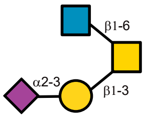 8%	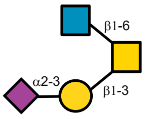 5%
1040	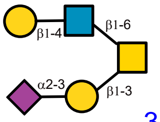 39%		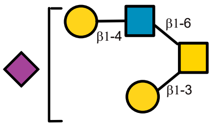 40%	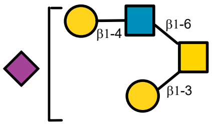 24%
1331	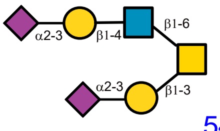 54%		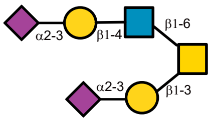 30%	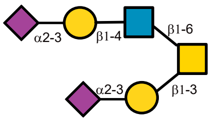 16%
829		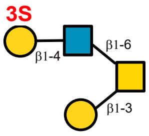 6%	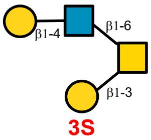 5%	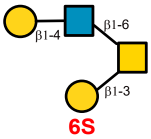 3%
1120		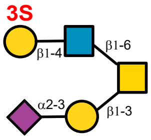 79%		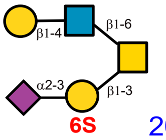 20%
599				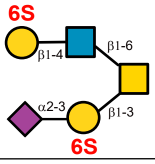 17%

Supplemental Figure 3 Table of glycans detected after transfection of CHO-cells with PSGL-1/mIgG2b and GCNT1 contructs together with constructs for sulfotransferases. Included is also the relative abundance as measured by their ion intensity of parent ions from the LC-MS runs. For key of symbols, see Figure 1.
